# Recent Advances in Prostate Cancer Treatment and Drug Discovery

**DOI:** 10.3390/ijms19051359

**Published:** 2018-05-04

**Authors:** Ekaterina Nevedomskaya, Simon J. Baumgart, Bernard Haendler

**Affiliations:** Therapeutic Research Groups, Research & Development, Pharmaceuticals, Bayer AG, Müllerstr. 178, 13353 Berlin, Germany; ekaterina.nevedomskaya@bayer.com (E.N.); simon.baumgart@bayer.com (S.J.B.)

**Keywords:** prostate cancer, androgen receptor, PI3K pathway, DNA repair, “omics” technologies

## Abstract

Novel drugs, drug sequences and combinations have improved the outcome of prostate cancer in recent years. The latest approvals include abiraterone acetate, enzalutamide and apalutamide which target androgen receptor (AR) signaling, radium-223 dichloride for reduction of bone metastases, sipuleucel-T immunotherapy and taxane-based chemotherapy. Adding abiraterone acetate to androgen deprivation therapy (ADT) in order to achieve complete androgen blockade has proven highly beneficial for treatment of locally advanced prostate cancer and metastatic hormone-sensitive prostate cancer (mHSPC). Also, ADT together with docetaxel treatment showed significant benefit in mHSPC. Ongoing clinical trials for different subgroups of prostate cancer patients include the evaluation of the second-generation AR antagonists enzalutamide, apalutamide and darolutamide, of inhibitors of the phosphatidylinositol-4,5-bisphosphate 3-kinase (PI3K) pathway, of inhibitors of DNA damage response, of targeted alpha therapy and of prostate-specific membrane antigen (PSMA) targeting approaches. Advanced clinical studies with immune checkpoint inhibitors have shown limited benefits in prostate cancer and more trials are needed to demonstrate efficacy. The identification of improved, personalized treatments will be much supported by the major progress recently made in the molecular characterization of early- and late-stage prostate cancer using “omics” technologies. This has already led to novel classifications of prostate tumors based on gene expression profiles and mutation status, and should greatly help in the choice of novel targeted therapies best tailored to the needs of patients.

## 1. Introduction

Despite recent diagnostic and therapeutic advances, prostate adenocarcinoma retains a high incidence in the male Western population and is responsible for about 20% of cancer-related deaths [1]. Numerous studies indicate that prostate cancer is driven by the androgen receptor (AR), a ligand-dependent transcription factor belonging to the nuclear receptor family [2,3]. In the absence of ligand (e.g., the major androgens dihydrotestosterone (DHT) and testosterone, or other androgenic steroids), the AR is located in the cell cytoplasm, complexed with chaperone proteins. After ligand binding, it translocates into the nucleus, and forms a homodimer following the interaction of dedicated motifs present in the DNA-binding domain (DBD) and the ligand-binding domain (LBD). In the cell nucleus, the dimerized AR recognizes cognate DNA response elements in regulatory regions located in proximal or more distal intra- and intergenic regions of androgen target genes [4,5]. It will then recruit a number of coregulator proteins and epigenetic factors to form a transcriptionally active complex and stimulate downstream gene expression [2,3]. Repression of gene activity following interaction with corepressors has also been described but is less well characterized [6]. Post-translational modifications such as phosphorylation, acetylation and ubiquitylation further fine-tune AR function [7,8]. Another level of complexity is added by the existence of several AR splice variants, some of which display constitutive, ligand-independent activity [9].

**Figure 1:**
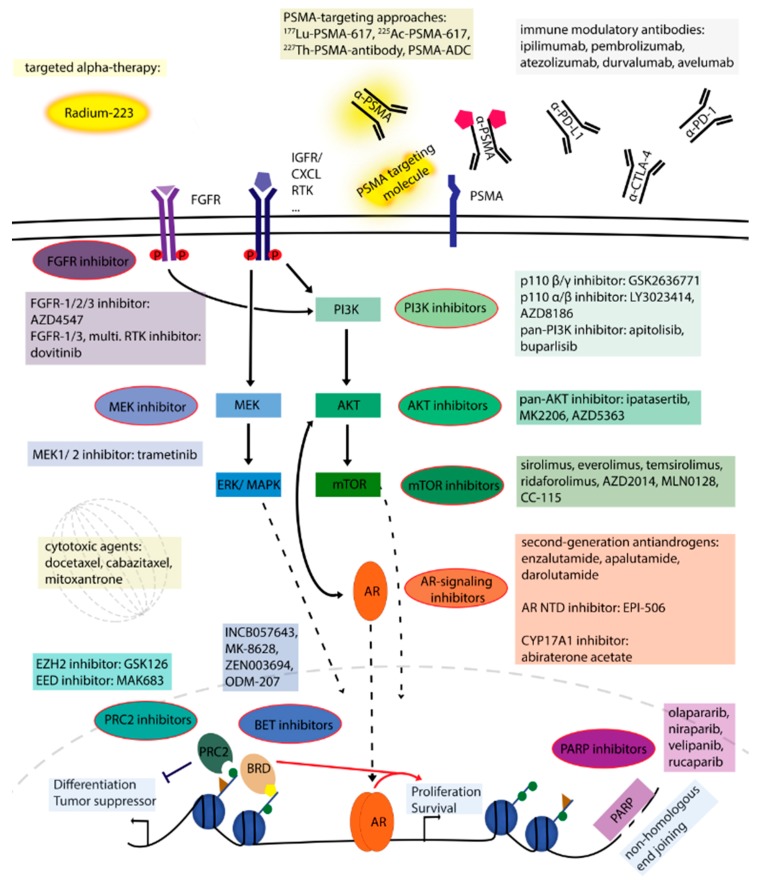
Crosstalk of androgen receptor (AR) signaling with other pathways, and selected inhibitors. Dotted lines were used for cytoplasm-to-nucleus signaling. The red arrow indicates the formation of a protein complex.

In nearly 90% of cases, prostate cancer is still organ-confined or only locally advanced at diagnosis [3]. Depending on multiple parameters which include clinical stage and prostate-specific antigen (PSA) levels, decisions are made whether to opt for active surveillance, local radiotherapy or prostatectomy [10]. Once the disease has spread outside the prostate, androgen deprivation therapy (ADT) by surgical or chemical castration to decrease circulating testosterone levels is often used [2,3]. Unfortunately, the response is only transient and most patients will develop resistance to ADT and progress towards castration-resistant prostate cancer (CRPC) after 18 to 36 months [3,9,11]. The AR axis is still an essential player in CRPC and therapeutic options to achieve maximal androgen deprivation by blocking AR function directly with competitive antagonists of the cognate ligand DHT or by reducing intra-tumoral androgen synthesis with a CYP17A1 lyase/hydrolase inhibitor have proven highly beneficial (Figure 1) [2,3,12]. Clinical studies are currently running with different second-generation AR antagonists given either as single agents or together with other drugs [13]. For metastatic CRPC (mCRPC), additional therapies including taxanes and the cell-based immunotherapy sipuleucel-T have been approved in recent years [3,11]. In addition, targeted alpha therapy with radium-223 dichloride has proven beneficial for the treatment of mCRPC with symptomatic bone metastases (Figure 1) [14]. Despite these advances, the disease will ultimately relapse and resistance mechanisms including AR gene amplification, LBD mutations and splice variants, and also local increase of androgen levels and altered levels of AR cofactors have been described, indicating that a more effective way of blocking AR signaling is likely to further improve the outcome [3,9,11,12].

Multiple growth-promoting and survival pathways interact with AR signaling and are involved in prostate cancer (Figure 1). The roles of the phosphatidylinositol-4,5-bisphosphate 3-kinase (PI3K)/Ak strain transforming (AKT)/mechanistic target of rapamycin (mTOR) [15,16] and DNA repair pathways [17,18] have been analyzed in much detail, and single-agent treatment with specific inhibitors as well as combination approaches with compounds interfering with AR signaling are currently being investigated in clinical studies [11]. Extensive alterations of epigenetic marks such as histone methylation and acetylation, and DNA methylation have been reported in prostate cancer [19,20,21]. Consequently, compounds addressing epigenetic targets such as the polycomb repressive complex 2 (PRC2) or acetyl mark-recognizing bromo- and extra-terminal (BET) proteins have entered clinical trials recently [22]. Finally, the impressive effects seen with immunotherapies in melanoma and non-small cell lung cancer [23] have led to the evaluation of immune checkpoint inhibitors in CRPC as well (Figure 1). Initial results with the cytotoxic T-lymphocyte-associated protein 4 (CTLA-4) monoclonal antibody ipilimumab have not met expectations [24]. Early clinical studies with agents targeting programmed cell death protein 1 (PD-1) or programmed death-ligand 1 (PD-L1) are currently ongoing.

Despite the fact that prostate cancer has one of the highest heritable risk of all cancers, the susceptibility loci identified so far only confer small risk increases [25]. Extensive efforts towards establishing the genomic landscapes of primary tumors and metastatic samples have revealed the strong intra- and inter-patient heterogeneity of prostate cancer and the multiple ontogenic changes the tumors undergo during their lifespan [26,27,28]. Aberrations in AR, E26 transformation-specific (ETS) family members, TP53, and phosphatase and tensin homolog (PTEN) are the most frequently detected in mCRPC [28,29], which suggests specific vulnerabilities of prostate cancer subgroups that may be amenable to dedicated targeted therapies. Also, novel classifications of prostate cancer based on gene expression signatures have been proposed [30,31,32]. These recent findings may greatly help deciding on the therapy most likely to benefit individual subgroups and represent a major step towards personalized medicine adapted to the individual needs of prostate cancer patients.

## 2. Treatment Options and Potential Novel Therapies

### 2.1. Compounds Targeting Androgen Signaling

The rising PSA levels measured in prostate cancer patients who become resistant to ADT indicate that AR signaling remains an essential target for pharmacological intervention at this stage. This vulnerability is addressed with steroid synthesis inhibitors and with second-generation AR antagonists (Figure 1). Selected phase 3 clinical studies with drugs targeting the AR pathway are listed in Table 1.

Abiraterone acetate is an irreversible inhibitor of CYP17A1, a 17–20 lyase and 17-α hydroxylase of the cytochrome P450 family, which converts pregnanes into steroid hormones, including androgen precursors [33]. It can, therefore, block androgen production in the testis and adrenal glands, and also in prostate tumors, thus preventing prostate cancer growth. The main side-effects originate from elevated mineralocorticoid levels consequent to CYP17A1 inhibition and are handled by concomitant prednisone or prednisolone treatment. Abiraterone acetate was first approved by the Food and Drug Administration (FDA) in 2011 for late-stage CRPC patients who have received docetaxel, and then in 2012 also for use prior to docetaxel therapy [33]. It is currently being tested together with several other therapies and very recently, the superiority of adding it to ADT in patients with locally advanced prostate cancer or mHSPC was evidenced in two independent clinical studies [34,35,36]. Additional CYP17A1 inhibitors such as orteronel and galeterone have been evaluated in clinical studies but did not reach their primary endpoint [37].

Enzalutamide is a second-generation, competitive oral AR antagonist approved by the FDA for mCRPC treatment post- and pre-docetaxel therapy in 2012 and 2014, respectively [38]. In addition, it strongly improves metastasis-free survival (MFS) in high-risk non-metastatic CRPC (nmCRPC) patients. Side-effects linked to brain penetration of the compound and including fatigue have been reported [39,40]. A clinical trial to assess the impact on cognitive function in elderly patients has been initiated [41]. In other ongoing clinical studies, enzalutamide is evaluated in combination with other drugs already approved for early- or late-stage prostate cancer such as abiraterone acetate, docetaxel or radium-223 dichloride [11]. Also, a phase 3 combination study with the PD-L1 antibody atezolizumab has been initiated [42]. Furthermore, numerous phase 1 and 2 clinical trials are assessing the combination of enzalutamide with various drugs targeting growth-promoting pathways and also with different immune checkpoint regulators (see below).

Apalutamide is an oral AR antagonist structurally related to enzalutamide [43] which has very recently gained approval for nmCRPC, based on a 24-month longer MFS [44]. Rash, fatigue, arthralgia, weight loss, fails and fracture were observed at higher rates, in comparison to the placebo group. Additional clinical trials are underway in prostate cancer patients where apalutamide is combined with GnRH ligands, abiraterone acetate, or the mTOR inhibitor everolimus. 

Darolutamide is a second-generation competitive oral AR antagonist with a novel chemical structure [45,46]. A strong binding to the AR LBD translating into potent anti-tumor efficacy in preclinical models and unique antagonistic activity against AR mutants identified in treatment-resistant prostate cancer patients have been reported [47,48]. Unlike other AR antagonists, darolutamide has low blood-brain barrier penetration, which may result in a better side-effect profile [47]. Phase 3 studies are presently ongoing for darolutamide in nmCRPC with MFS as the primary endpoint and for darolutamide in addition to standard ADT plus docetaxel in mHSPC.

A strategy to bypass resistance linked to LBD mutations is to target other potentially druggable AR regions. In one approach, non-competitive AR antagonists binding to the N-terminal domain have been considered. A compound family that binds to the Tau-5 region of the AR N-terminal domain and prevents interaction with cofactors and downstream gene activation has been described [49]. The most advanced compound is ralaniten acetate (EPI-506) which was tested in clinical phase 1/2 for mCRPC [50]. Other approaches to block AR signaling deal with the binding function 3 located at the LBD surface and with a short region of the DBD for which small molecule inhibitors with in vitro or in vivo efficacy in several prostate cancer models have been identified [51,52]. 

### 2.2. Signaling Pathway Inhibitors

The essential role of the PI3K/AKT/mTOR pathway in prostate cancer initiation and progression has been documented by numerous studies [15,16]. PTEN loss is a key event responsible for hyperactive PI3K signaling and is associated with negative outcomes in prostate cancer patients [53]. A genetically engineered mouse model with prostate-specific deletion of the *Pten* gene recapitulates the different disease stages observed in humans [54]. The p110β subunit of PI3K appears to be the main player but its blockade results in upregulation of the p110α subunit so that combined blockade of both isoforms is more effective to prevent prostate cancer growth in PTEN null mice [55]. Also, a crosstalk and reciprocal feedback mechanism between the PI3K/AKT/mTOR pathway and AR signaling have been reported [56]. Inhibition of the PI3K pathway increases AR protein levels and partly restores AR signaling [56]. Conversely, AR inhibition reduces the levels of the AKT dephosphorylating enzyme PH domain and leucine-rich repeat protein phosphatase, thereby enhancing AKT signaling [56,57]. Treatment with inhibitors of the PI3K/AKT/mTOR pathway shows in vivo anti-tumor efficacy in different preclinical models [58,59,60] and concomitant AR antagonist application gives superior benefit [56,61]. Disappointingly, first clinical studies with agents interfering with the PI3K/AKT/mTOR pathway have only shown limited efficacy [62,63,64]. The most advanced compound is the AKT inhibitor ipatasertib [65] which is currently in clinical phase 3 for mCRPC patients. Additional clinical evaluations are currently ongoing (Table 2) and will hopefully be more successful. In some of these trials, combinations with abiraterone acetate or enzalutamide are also being evaluated, but here also first phase 2 results have not looked very promising [15]. Patient stratification based on PTEN loss or mutations in the PI3K/AKT/mTOR pathway may lead to better outcomes [66]. Importantly, such alterations have been reported in a sizeable subgroup of prostate cancer patients, in primary tumors and more so in metastases (see below).

The limited response to PI3K/AKT/mTOR inhibitors may be linked to compensatory activation of the mitogen-activated protein kinase (MAPK) cascade and experiments with preclinical prostate cancer models show improved benefit following a combined blockade of both pathways [67,68]. Clinical studies are currently ongoing with trametinib, an inhibitor of MAPK kinase (MEK) which is upstream in the MAPK pathway, in late-stage prostate cancer.

Fibroblast growth factor (FGF) signaling also plays a role in prostate cancer, especially in late-stage disease [69,70]. Treatment of prostate cancer xenografts with the FGF receptor inhibitor AZD4547 reduces tumor growth, and the effects are more pronounced when additionally treating with AZD5363, an AKT inhibitor [71]. Several clinical studies are underway with AZD4547, but there is no dedicated trial addressing prostate cancer. Dovitinib is an FGF receptor kinase inhibitor which additionally blocks other receptors. A phase 2 study in mCRPC showed only limited efficacy [72].

Another pathway with an essential role in prostate cancer is wingless/integrated (WNT) signaling, for which both autocrine and paracrine mechanisms have been implicated [73]. Preclinical data show the involvement of both canonical and non-canonical WNT signaling in prostate cancer. A tumor-suppressive role has also been described for some members of the WNT pathway and early clinical studies are currently ongoing in different indications including metastatic prostate cancer to evaluate a peptide mimetic of WNT-5A which showed promising results in an orthotopic prostate cancer mouse model [74]. Deregulation of both the PI3K and WNT pathways further enhances prostate tumor growth due to aberrant downstream mTOR signaling [75]. The occurrence of mutations in the WNT pathway is low in prostate cancer so that recent studies have focused on the non-canonical WNT pathway, which includes additional players and is involved in tumor migration and invasion [76]. Interestingly, a genome-wide analysis of patients with primary resistance to abiraterone acetate revealed frequent aberrations in the WNT pathway [77]. First compounds interfering with WNT signaling at different levels have reached the clinic but no efficacy results are yet available [73].

### 2.3. DNA Damage Repair Pathway

DNA damage response (DDR) defects have a comparatively high incidence in advanced prostate cancer patients and include mainly mutations in the homologous recombination and DNA mismatch repair pathways [17]. Initial efforts to address this liability in the clinic focused on blocking the poly ADP ribose polymerase (PARP) family, which plays a key role in detecting damaged DNA and assisting repair [17,18]. Olaparib is an oral PARP inhibitor which was evaluated in mCRPC patients and positive phase 2 data were reported, with anemia and fatigue being the most observed side-effects [78]. Importantly, patients with mutated breast cancer susceptibility (BRCA) 1 or 2 or mutated ataxia telangiectasia serine/threonine kinase (ATM), which are all essential players in DNA repair mechanisms, generally show a better response [78]. Other recent findings suggest that loss of the chromatin remodeler chromodomain-helicase-DNA-binding protein 1 (CHD1), which is often observed in advanced prostate cancer, increases the response to PARP inhibitors [79,80]. The encouraging clinical phase 2 results obtained with olaparib led to a breakthrough designation by the FDA and a phase 3 pivotal study is currently ongoing [81]. Conversely, the clinical results obtained for the PARP inhibitor veliparib given to mCRPC patients in addition to abiraterone acetate did not show a statistically significant improvement [82]. Prostate cancer clinical studies evaluating drugs targeting PARP are listed in Table 3.

Another approach is the inhibition of ataxia telangiectasia and Rad3-related kinase (ATR), which senses single-strand DNA breaks [83]. Potent anti-tumor efficacy was observed for the ATR inhibitor BAY 1895344 in combination with radium-223 dichloride in a bone metastasis xenograft model of CRPC [84]. Chk1 is a cell-cycle regulator of DNA damage response downstream of ATR and recent data show its inhibitor AZD7762 to be additive or synergistic with enzalutamide in prostate cancer xenografts [85]; however clinical studies with this compound have been discontinued. 

### 2.4. Epigenetic Mechanisms

Dissection of the function of PRC2 shows that it epigenetically represses the expression of tumor suppressor genes essential for prostate cancer initiation and progression [86]. A PRC2 component is enhancer of zeste 2 (EZH2) which trimethylates lysine 27 of histone H3 to repress transcription and its gene is one of the most consistently overexpressed ones in metastatic prostate cancer [87,88]. The role of EZH2 in prostate cancer progression has been reported in different studies [89,90]. Selective, potent EZH2 inhibitors have recently been identified and anti-proliferative activity was reported in prostate cancer cell lines for GSK126 and derivatives [91,92]. This was however not seen when using EZH2 inhibitors with another chemical scaffold [93]. EED is another core component of the PRC2 complex and its inhibitor MAK683, which is related to EED226 [94], has recently entered a clinical phase 1 study which includes prostate cancer patients [95].

Another histone methyltransferase complex involved in prostate cancer is the mixed lineage leukemia (MLL) complex which is responsible for histone H3 lysine 4 methylation. Importantly, potential driver mutations in *MLL* gene family members have been identified in prostate cancer samples by exome sequencing analysis [28,96]. A direct interaction between the MLL complex and the AR was reported [96,97] and an inhibitor of menin, which is part of the MLL complex, prevents the growth of prostate cancer xenografts [97].

LSD1 is a histone demethylase found to interact with the AR and to be important for its function [98]. It is involved in prostate cancer progression by activating a subgroup of cell-cycle genes [99]. The identification of selective LSD1 inhibitors allowed the delineation of its role in different tumor types, including prostate cancer [100]. Recent findings based on the allosteric inhibitor SP-2509 indicate that the demethylase activity of LSD1 is not needed to promote prostate cancer growth [101]. The first LSD1 inhibitors have entered the clinic for various cancer indications [102]. 

The BET family members possess dual bromodomains which recognize histone acetylation marks and are essential for the formation and activity of transcriptional elongation and super-enhancer complexes [103,104,105]. Their role in prostate cancer was evidenced following the identification of selective, potent inhibitors [22,104]. Strong in vivo efficacy was reported for BET inhibitors with various chemical scaffolds and also for proteolysis targeting chimera (PROTAC) derivatives in different prostate cancer xenografts and in patient-derived models [106,107,108,109,110]. Interestingly, resistance mechanisms to BET inhibitors linked to mutations in the speckle-type POZ protein (SPOP), a component of the cullin-RING-based E3 ubiquitin ligase complex, which result in BET protein stabilization, and to CDK9-mediated phosphorylation of AR, have been identified [111,112,113]. Clinical trials are ongoing in solid tumors for several BET inhibitors including MK-8628, ZEN003694, INCB057643 and ODM-207 [22,95,114].

CBP/p300 are two related histone acetyltransferases with essential coactivator functions for several transcription factors including the AR [115]. The recent identification of inhibitors of CBP/p300 enzymatic activity or of their bromodomain has greatly helped to outline the implication of these paralog proteins in prostate cancer. A-485 is an inhibitor of CBP/p300 acetyltransferase activity which impairs AR function in prostate cancer cells and shows in vivo efficacy in a castration-resistant model [116]. GNE-049 is a specific inhibitor of the CBP/p300 bromodomain which also blocks AR function and exhibits anti-tumor efficacy in several patient-derived prostate cancer models [117].

### 2.5. Targeted Alpha Therapy Approach

Radium-223 dichloride is a targeted alpha therapy administered intravenously. It mimics calcium and is selectively taken up in osteoblastic bone metastases and binds to hydroxyapatite, a major component of bone [118,119]. In vivo experiments performed in prostate cancer xenograft models show that once deposited in the newly formed intra-tumoral bone matrix, radium-223 dichloride has cytotoxic effects on adjacent tumor cells, osteoclasts and disease-promoting osteoblasts by inducing difficult-to-repair DNA double-strand breaks, thereby disrupting positive feedback loops between tumor microenvironment cells and osteoblasts [120]. Preclinical evidence for synergistic effects with the ATR inhibitor BAY 1895344 has been reported [84].

When given to CRPC patients, radium-223 dichloride significantly improves overall survival irrespective of prior docetaxel use, while alleviating pain linked to bone metastases and with a favorable safety profile [121,122,123]. It was originally approved by the FDA in 2013 for CRPC patients with bone metastases and no known visceral metastatic disease [14,124]. A clinical study in which radium-223 dichloride was combined with abiraterone acetate and prednisone was unblinded early due to increased death and fracture risk, but other combination studies with enzalutamide, olaparib and niraparib are ongoing (Table 4) [122]. Also, recent data show that radium-223 dichloride induces T cell-mediated lysis in different tumor types including prostate cancer [125], so that combining this drug with immunotherapies may have additional clinical benefit. Indeed, early combination studies for radium-223 dichloride and different checkpoint inhibitors including pembrolizumab and atezolizumab are ongoing (Table 4). 

### 2.6. Prostate-Specific Membrane Antigen (PSMA) Targeting Approaches 

Prostate-specific membrane antigen (PSMA) is highly expressed on prostate cancer cell membranes and its levels are increased following treatment with abiraterone acetate or enzalutamide [126]. Specific antibody-drug conjugates (ADCs) targeting PSMA and loaded with cytostatic agents such as auristatin or maytansinoid have been described [127]. Studies with patient-derived prostate cancer xenografts have shown strong efficacy of such derivatives but also high response variability [128]. First clinical studies with a PSMA-targeting ADC gave positive results [127] but no pivotal phase 3 study has yet been initiated. Importantly, the combination of abiraterone acetate or enzalutamide with PSMA-targeting ADCs shows synergistic effects in preclinical prostate cancer models [129].

PSMA-targeting small molecule ligands or antibodies labeled with radionuclides have been evaluated in several clinical studies. Radioligand therapy using the beta-emitter lutetium-177 had encouraging efficacy results with limited toxicity in patients with advanced prostate cancer so that randomized clinical trials are currently ongoing with lutetium-177-PSMA-617 [130]. Targeted alpha therapy with actinium-225-PSMA-617 shows even stronger anti-tumor effects in patients, based on surrogate parameters, but the therapeutic range needs to be improved [131]. A novel alpha therapy approach deals with a thorium-227-labeled PSMA antibody. It shows strong in vitro potency in several PSMA-positive cell lines and in vivo efficacy in xenograft models of prostate cancer [132]. 

### 2.7. Chemotherapy

Different chemotherapeutic drugs are approved for prostate cancer treatment. Docetaxel and cabazitaxel are given intravenously every three weeks to prevent tubulin depolymerization and thereby mitotic cell division, eventually leading to cell death [133]. In addition, AR inhibitory properties linked to the prevention of microtubule-dependent nuclear transport have been proposed [134]. Docetaxel and cabazitaxel were approved by the FDA for mCRPC in 2004 and 2010, respectively [135,136]. Docetaxel was also evaluated in mHSPC together with ADT and positive results were reported in two different clinical trials [137]. Hematological toxicity such as neutropenia is a frequently observed adverse effect. 

Mitoxantrone is a topoisomerase inhibitor with limited survival benefit in prostate cancer patients compared to docetaxel but with some symptomatic relief [138]. Combination with cabazitaxel leads to durable responses in chemonaïve mCRPC patients [139]. The use of mitoxantrone is, however, limited by significant adverse events [140].

### 2.8. Immunotherapy

Sipuleucel-T was approved in 2010 for mCRPC [141]. For this autologous cellular immunotherapy, peripheral blood mononuclear cells from the patient are prepared by leukapheresis and then cultured in the presence of recombinant prostatic acid phosphatase (PAP) coupled to granulocyte-macrophage colony-stimulating factor for maturation of antigen-presenting cells. The activated product is then returned to the patient in three courses of intravenous infusions with the aim to fight cancerous prostate cells that express high levels of PAP. Long-term clinical benefits with only mild, manageable side-effects have been reported [141]; however, the high costs and complex procedure have limited the use of sipuleucel-T until now [142,143].

Prostate cancer has a comparatively low proportion of tumor-specific neoantigens, suggesting that it may be less likely to respond to treatments addressing immune checkpoint inhibitors [144,145]. On the other hand, defects in DNA repair pathways have been reported both in early- and late-stage patients and may increase neoantigen burden [17]. Another concern is the prostate tumor microenvironment which is immunosuppressive and impairs natural killer cell function [146]. Also, studies performed mainly in non-tumor models suggest that local sex steroids may influence immune cells in the prostate microenvironment [147]. Nonetheless, in view of the strong survival advantage provided by therapies with immune checkpoint inhibitors in different cancer types, the potential benefits of immunotherapy are also being evaluated in prostate cancer [42,148]. First studies with the anti-CTLA4 antibody ipilimumab showed some promising results in early clinical evaluations addressing prostate cancer patients, but no improved overall survival could be determined afterwards in larger studies [24], even though complete remission was observed in a few cases [149]. The anti-PD-1 antibody pembrolizumab is being investigated in two clinical trials and durable responses have already been reported in patients with metastasized prostate cancer [42,148]. Importantly, pembrolizumab recently received a tissue-agnostic approval for mismatch repair-deficient solid tumors, so that advanced prostate cancer patients belonging to this group can also be treated with this drug [150]. Concerning anti-PD-L1 antibodies, early clinical studies are ongoing with atezolizumab, durvalumab and avelumab for treatment of metastatic prostate cancer [42]. The most advanced trial compares atezolizumab as single agent to a combination with enzalutamide in a randomized phase 3 study [42]. Possibly, selecting patients based on tumor PD-L1 expression or on defects in DNA repair pathways, or combining with compounds that increase genomic instability could improve the outcome of studies testing such checkpoint inhibitors [42,148]. An extensive overview of prostate cancer clinical trials evaluating this biological compound group has recently been published [42].

## 3. Advances in Molecular Characterization of Prostate Cancer through “Omics” Technologies

### 3.1. Primary Prostate Cancer

As mentioned above, 90% of prostate cancer patients have localized disease at the time of diagnosis [10]. The course of their clinical response to therapy varies greatly, which suggests substantial molecular heterogeneity of primary prostate cancer. Advances in next-generation sequencing (NGS) technologies have brought opportunities to classify this heterogeneous disease into sub-groups characterized by differences in the genomic, transcriptomic and epigenetic make-up of tumors. Understanding the underlying molecular alterations can guide treatment choice, improve prediction of patients’ outcome and reveal new biomarkers and therapy targets.

One of the characteristic genomic features of prostate cancer is the low occurrence of mutations and focal copy number events, while the rate of broad copy number aberrations and gene fusions is high [151]. The most common alterations in prostate cancer are fusions of *ETS* gene family members with androgen-regulated genes. These fusions usually occur early in prostate cancer development and result in overexpression of the fused *ETS* family gene [152]. A notable and well-described example is the fusion between the androgen-controlled transmembrane protease, serine 2 (*TMPRSS2*)gene and the ETS-related gene (*ERG*), which is found in around 50% of the tumors [153]. The high frequency of this fusion and its seemingly driving role in the initiation of prostate cancer makes it a potentially attractive therapeutic target. On the other hand, *ERG* overexpression was reported to be a favorable prognosis factor in the context of wild-type *PTEN* [154]. Addressing transcription factors such as ERG is challenging, due to the absence of druggable regions. Some attempts have been made in identifying specific inhibitory peptides [155] and small molecules [156] to target ERG and/or the fusion. In vitro and in vivo efficacy in prostate cancer models with the ERG fusion was reported for some compounds but no clinical candidate has been identified yet. Four out of seven molecular subtypes of primary prostate cancer defined in the large Cancer Genome Atlas study of 333 tumors harbor fusion or overexpression of one of the *ETS* family genes, namely *ERG*, *ETV1*, *ETV4* or *FLI1* [27]. The other three molecular classes identified in this study are characterized by mutations in *SPOP*, *FOXA1* or isocitrate dehydrogenase 1 (*IDH1*) genes. Importantly, mutations in these genes, as well as *ETS* gene fusions, are mostly mutually exclusive, which corroborates the existence of distinct molecular mechanisms leading to these prostate cancer subtypes. A number of coexisting alterations in known cancer genes were reported as well. Mutations and deletions in genes from the *PI3K* pathway (e.g., *PIK3CA*, *PTEN*) and *TP53* mutations are often concurrent with *ETS* gene fusions, while deletion of chromodomain-helicase *CHD1* is predominantly observed in the *FOXA1* and *SPOP* subclasses [157]. Such prostate cancer classification systems with clear molecular basis will hopefully assist clinical prognosis of patients and decision-making for therapy in the near future.

Importantly, structural changes in the genome, such as fusion of *ETS* family genes, translate into marked changes at other functional genomic levels. The *TMPRSS2*–*ERG* fusion status of tumors is associated with significant differences in DNA methylation profiles [158], cistrome (e.g., histone acetylation H3K27ac) and transcriptome [159].

There are a number of alternative efforts to classify prostate cancer. They go beyond defining driving genetic lesions, but rather take inspiration from breast tumors and other cancer types, where distinct transcriptional profiles exist and are used in prognostication and treatment selection. Recently the well-known PAM50 gene expression classifier, used for identification of breast cancer molecular subtypes, was applied to prostate cancer [31]. Given the similarities in the role of steroid hormones in prostate and breast cancer and the many shared oncogenic pathways, it is perhaps not surprising that this signature can also robustly classify prostate cancer. Three groups of prostate tumors were defined using the PAM50 classification: luminal A, luminal B and basal. They show differences in clinical prognosis, independently of known clinico-pathological parameters. Other transcriptomics classification efforts, although based on a different method, also consistently distinguish between luminal and basal groups [30]. The association of hormonal signaling and prostate cancer classification is of particular interest for predicting patients’ response to ADT and/or selecting patients for adjuvant therapy.

### 3.2. Advanced Prostate Cancer

Despite the recent advances made, metastatic prostate cancer remains deadly. Characterizing the genomic landscape of advanced prostate cancer can lead to the discovery of new avenues for treatment. Except in the rare cases where neuroendocrine differentiation is observed [160,161], prostate cancer that progressed after ADT to CRPC remains dependent on androgen signaling. This explains the high frequency of AR aberrations (amplification and mutations) that are exclusive to CRPC and not seen in primary tumors [162]. Additionally, the expression of a number of AR cofactors, transcriptional coactivators and chromatin modifiers is also altered in CRPC, which requires next-generation RNA sequencing for precise analysis [26,96]. Genomic technologies deepened our understanding of the biology of lethal prostate cancer and allowed the identification of a number of actionable alterations enriched in CRPC. These include aberrations in the PI3K/AKT/mTOR pathway and in DDR. Indeed, new treatment regimens for CRPC patients with drugs targeting these pathways are already being evaluated (see above).

An intriguing recent observation suggests that the increasing use of potent anti-androgens and inhibitors of androgen synthesis (enzalutamide and abiraterone acetate, respectively) in the management of advanced prostate cancer promotes development of so-called double-negative CRPC [32]. This group is independent of androgen signaling and does not display neuroendocrine characteristics. Although based on a limited number of patients, genomic analysis indicates that progression into this double-negative phenotype is not related to the acquisition of alterations in known prostate cancer genes. These tumors often exhibit active FGF receptor and MAPK signaling to bypass the AR pathway for sustained prostate cancer growth, and initial preclinical experiments with selective inhibitors show in vitro and in vivo efficacy in double-negative prostate cancer models [32].

Finally, subtypes of prostate cancer bone metastases have also been identified by expression profiling [163]. Here, two groups, one with high AR and metabolic activity, and low immune response, and one with the opposite features, were described. Altogether, these novel molecular classification systems for stratification of advanced prostate cancer subgroups have the potential to greatly support the treatment choice for patients, with the ultimate goals to improve both overall survival and quality of life.

### 3.3. Clonal Evolution and Dynamics

In addition to assessing inter-tumor heterogeneity, NGS data provide details on clonality of genetic alterations within a tumor, informing on the evolutionary route of a particular cancer, its founding, earliest occurring genetic alterations, as well as the presence of clones that can lead to resistance to therapy [164]. The recent study of whole-genome sequences of 57 primary prostate tumors highlighted *NKX3-1* and *FOXP1* loss, and *TMPRSS2–ERG* fusion as highly clonal, meaning that these events occur very early in tumor development and are probably important for tumor initiation [165]. In contrast, *PTEN* loss, lesions in *TP53* and *CDKN1B* were subclonal, which suggests their accumulation in later stages of cancer progression [165]. Understanding clonality of mutations can help improve prognostication and predict therapy response in cancer patients [166].

NGS analysis of multiple longitudinal samples from the same patient allows not only to reconstruct the sequence of events in primary tumor development, but also to better understand evolution towards metastatic disease, and also how seeding of metastases occurs and therapy resistance arises. A couple of studies on a few patients have shown that lethal metastases can arise from very small, not dominant clones present in the primary tumor [167]. In the evolutionary reconstruction of lethal prostate cancers a number of genetic lesions (notably the *TMPRSS2–ERG* gene fusion) are present in the “trunk” of the evolutionary tree and are carried by metastases as genetic fingerprints of their origin [168]. The same work additionally brought evidence for metastasis-to-metastasis spread. In another study, multiple independent clones were detected in metastatic prostate cancer and the emergence of AR mutations leading to resistance could be temporally linked to abiraterone acetate treatment [169]. Dissecting founding mutations, as well as understanding which mutations are responsible for metastatic dissemination, will hopefully lead to therapy regimens that eliminate tumors and reduce the likeliness of developing lethal metastasis.

Liquid biopsies for access to circulating tumor DNA (ctDNA) and circulating tumor cells (CTCs) allow non-invasive detection of somatic mutations and follow-up of therapy response of cancer patients. They have improved the diagnosis and treatment of prostate cancer in recent years [170]. *AR* gene copy number changes and mutations before or during therapy can thereby be monitored [169]. Detection of the *AR-V7* splice variant was linked to poorer response to AR-targeting drugs [171]. Also, the expression of enzymes involved in steroid synthesis was monitored in CTCs [172].

### 3.4. Proteomics and Metabolomics

While the advances of genomic technologies greatly expand our knowledge of the genetic basis of prostate cancer, one should not overlook the fact that proteins are the essential effectors of all genetic processes and that the vast majority of drug targets are proteins. Broad quantitative proteomic profiling of a number of prostate cancer tumors has been performed and has allowed detection of implicated proteins such as proneuropeptide-Y [173]. Comparative analysis of malignant and benign prostate tissues showed deregulation of a few processes, notably increased oxidative phosphorylation capacity in cancer and a decrease in the abundance of proteins involved in cell adhesion. Identifying such differences can lead to novel targetable vulnerabilities for the management of prostate cancer. As part of the Cancer Genome Atlas efforts, targeted proteomic analysis of prostate tissues was performed using the reverse phase protein assay (RPPA). Distinct tumor clusters were identified but the comparison of activated pathways detected by RPPA and the presence of activating mutations in the same pathways found in genomics analyses showed only limited correlation [27].

An important feature of normal prostate epithelium is its unconventional, inefficient cellular metabolism that bypasses oxidative phosphorylation [174]. Primary prostate cancer switches to a more efficient energy production by restoring oxidative phosphorylation, which is again reduced in advanced cancer. This makes prostate cancer a “metabolic outlier” among other tumors and an attractive subject to study with metabolomics approaches. Metabolite-measuring techniques, such as nuclear magnetic resonance, have been successfully applied to differentiate benign from malignant prostate tissue since the mid-eighties [175,176]. More recent studies have focused on profiling biofluids for identification of biomarkers of the disease with a potential to improve current screening and detection of prostate cancer (for a comprehensive list see [177]). While such approaches are very attractive due to their non-invasive nature, the utility of these biomarkers for diagnosis is still to be seen.

## 4. Conclusions and Perspectives

Major scientific advances have increased the arsenal of drugs available to treat advanced prostate cancer in recent years. Efforts are now underway to investigate the efficacy of these compounds in earlier stages of the disease and also to combine and use them sequentially in patients. ADT plus abiraterone acetate or docetaxel treatment prolongs overall survival in mHSPC patients [137]. This additional docetaxel therapy had, however, no impact on the subsequent use of abiraterone acetate or enzalutamide [136]. A major recent finding came from two separate clinical trials which demonstrated that abiraterone acetate added to ADT had a major benefit for early stage patients [34,35,36]. Conversely, successive treatment of CRPC patients with abiraterone acetate followed by enzalutamide or vice-versa showed little additional benefit, suggesting common resistance mechanisms centered on AR signaling take place [178]. These data have led to new guideline recommendations for the management of prostate cancer, but additional prospective and randomized studies are needed to optimize the sequence and combination of approved drugs in order to come up with the best treatment strategy tailored to each patient’s needs [178].

Despite these advances, there is still a high medical need for additional therapy options. In the vast majority of cases, the AR remains the main driver in CRPC and novel second-generation AR antagonists with improved efficacy to overcome resistance mechanisms and with reduced side-effects are in late-stage clinical testing [2,13]. Also, novel strategies to target different AR domains are being evaluated [50,51,52]. In addition, other pathways with an essential role in prostate cancer growth are being addressed. Inhibitors of the PI3K/AKT/mTOR pathway and of DDR are currently in advanced clinical trials for different prostate cancer populations, either as single agents or in combination with other drugs [15,16]. 

Pivotal clinical studies with the CTLA-4 antibody ipilimumab did not reach their primary endpoint in mCRPC [42,148]. Trials are now ongoing with PD-1 and PD-L1 antibodies but it is too early to predict their outcome. In view of the low neo-antigenicity and poor lymphocyte infiltration of prostate tumors, strategies to enhance prostate tumor immunogenicity prior to therapy may be needed [179,180]. Also, novel clinical study designs tailored to immunotherapies with regard to treatment duration, endpoints, response assessment and patient selection may be necessary to increase the likeliness of success [148].

Selecting the optimal drug, or drug sequence and combination for prostate cancer treatment will be greatly aided by the identification of molecular biomarkers predictive of response and progression. Major advances have recently been made in the isolation and analysis of liquid biopsies [170]. CTCs and ctDNA were successfully used for the analysis of genetic and epigenetic alterations using NGS of whole exome DNA and by the determination of transcribed coding and non-coding RNA profiles [171,172,181]. Indeed, clinical studies evaluating the impact of the most frequent prostate cancer genetic alterations such as the *TMPRSS2–ERG* fusion, the truncated *AR-V7* splice variant, the *PTEN* deletion and DNA repair defects on treatment resistance have already generated first results [178]. In addition, a classification system based on genome wide transcriptome profiles has emerged in recent years. Like in breast cancer, a subdivision in luminal A, luminal B and basal subtypes with different clinical prognoses and responses to ADT has been proposed for prostate cancer [30,31]. Such a classification may greatly support treatment choice for early- and late-stage disease and ultimately improve the overall survival rate and quality of life of prostate cancer patients.

## Figures and Tables

**Table 1:** Selected phase 3 clinical studies in prostate cancer with drugs addressing the AR pathway. Source: https://clinicaltrials.gov/.

Agents	Population	Status	Identifier
ADT +/− abiraterone acetate + prednisolone ADT +/− abiraterone acetate + prednisolone + enzalutamide	Advancing or metastatic castration-resistant prostate cancer (mCRPC)	Completed	NCT00268476
Abiraterone acetate + prednisone/prednisolone	mCRPC post-chemotherapy	Completed	NCT00638690
Abiraterone acetate + prednisone	Asymptomatic or mildly symptomatic patients with mCRPC	Completed	NCT00887198
ADT +/− abiraterone acetate + prednisone	Metastatic hormone-naive prostate cancer	Active, not recruiting	NCT01715285
ADT + docetaxel +/− local radiation therapy +/− abiraterone acetate + prednisone	Metastatic hormone-naive prostate cancer	Recruiting	NCT01957436
Enzalutamide +/− abiraterone acetate + prednisone	mCRPC	Active, not recruiting	NCT01949337
Enzalutamide	nmCRPC	Active, not recruiting	NCT02003924
Enzalutamide +/− radium-223	Castration-resistant prostate cancer (CRPC)	Recruiting	NCT02194842
Enzalutamide + ADT	Metastatic prostate cancer	Active, not recruiting	NCT02446405
Enzalutamide + ADT + radiation therapy	High-risk localized prostate cancer	Recruiting	NCT02446444
Apalutamide	nmCRPC	Active, not recruiting	NCT01946204
Abiraterone + prednisone +/− apalutamide	Chemotherapy-naive mCRPC	Active, not recruiting	NCT02257736
ADT +/− apalutamide	mHSPC	Active, not recruiting	NCT02489318
Darolutamide	nmCRPC	Recruiting	NCT02200614
ADT + docetaxel +/− darolutamide	mHSPC	Recruiting	NCT02799602

**Table 2:** Selected clinical studies focusing on prostate cancer with drugs addressing the PI3K/AKT/mTOR pathway. Source: https://clinicaltrials.gov/.

Agents	Target	Population	Phase	Status	Identifier
Buparlisib	PI3K	High-risk prostate cancer	2	Active, not recruiting	NCT01695473
AZD8186 +/− abiraterone acetate + prednisone	PI3Kβ/δ CYP17A1	Advanced CRPC	1	Recruiting	NCT01884285
GSK2636771 + enzalutamide	PI3Kβ AR	mCRPC	1	Recruiting	NCT02215096
Enzalutamide +/− LY3023414	AR PI3K/mTOR	mCRPC	2	Recruiting	NCT02407054
Apitolisib or ipatasertib +/− abiraterone acetate + prednisone/prednisolone	PI3K AKT CYP17A1	CRPC post-docetaxel	1/2	Active, not recruiting	NCT01485861
Ipatasertib +/− abiraterone acetate + prednisone/prednisolone	AKT CYP17A1	mCRPC	3	Recruiting	NCT03072238
MK2206	AKT	Recurrent prostate cancer	1	Active, not recruiting	NCT01480154
Bicalutamide +/− MK2206	AR AKT	Recurrent prostate cancer	2	Active, not recruiting	NCT01251861
Docetaxel +/− AZD5363	tubulin AKT	mCRPC	2	Recruiting	NCT02121639
Enzalutamide +/− AZD5363	AR AKT	mCRPC	2	Recruiting	NCT02525068
Sirolimus + docetaxel + carboplatin	mTOR tubulin DNA	mCRPC	1/2	Recruiting	NCT02565901
Ridaforolimus	mTOR	Taxane-resistant androgen-independent prostate cancer	2	Completed	NCT00110188
Ridaforolimus + bicalutamide	mTOR AR	Prostate cancer	2	Completed	NCT00777959
Temsirolimus	mTOR	High-risk prostate cancer	2	Completed	NCT00071968
AZD2014	mTOR	High-risk prostate cancer	1	Active, not recruiting	NCT02064608
MLN0128	mTOR	Advanced CRPC	2	Active, not recruiting	NCT02091531
Temsirolimus + cixutumumab	mTOR IGF-1R	mCRPC	1/2	Completed	NCT01026623
Everolimus + docetaxel	mTOR tubulin	mCRPC	1/2	Completed	NCT00459186
Everolimus + bevacizumab	mTOR VEGF	Advanced prostate cancer	1/2	Completed	NCT00574769
Everolimus + carboplatin	mTOR DNA replication	mCRPC post-docetaxel	2	Completed	NCT01051570
Everolimus + radiation therapy	mTOR	Prostate cancer	1	Recruiting	NCT01548807
CC-115 + enzalutamide	DNA-PK/ mTOR AR	CRPC	1	Recruiting	NCT02833883

**Table 3:** Selected clinical studies focusing on prostate cancer with drugs targeting PARP. Source: https://clinicaltrials.gov/.

Agents	Target	Population	Phase	Status	Identifier
Olaparib	PARP	High-risk prostate cancer	2	Recruiting	NCT03047135
Olaparib + abiraterone acetate + prednisone/prednisolone	PARP CYP17A	mCRPC	2	Active, not recruiting	NCT01972217
Olaparib +/− degarelix	PARP GnRH antagonist	Intermediate/high-risk prostate cancer	1	Recruiting	NCT02324998
Olaparib +/− cediranib	PARP VEGFR	mCRPC	2	Recruiting	NCT02893917
Olaparib +/− abiraterone acetate + prednisone vs. abiraterone acetate + prednisone	PARP CYP17A	mCRPC	2	Recruiting	NCT03012321
Olaparib + radium-223	PARP Hydroxyapatite	mCRPC	1/2	Not yet recruiting	NCT03317392
Veliparib +/− temozolomide	PARP Rapidly dividing cells	mCRPC	1	Completed	NCT01085422
Abiraterone acetate + prednisone +/− veliparib	CYP17A PARP	mCRPC	2	Active, not recruiting	NCT01576172
Rucaparib	PARP	mCRPC	2	Recruiting	NCT02952534
Rucaparib vs. abiraterone acetate + prednisone or enzalutamide or docetaxel	PARP CYP17A AR Tubulin	mCRPC with homologous recombination gene deficiency	3	Recruiting	NCT02975934
Niraparib	PARP	mCRPC and DNA repair anomalies	2	Recruiting	NCT02854436
Niraparib + radium-223	PARP Hydroxyapatite	CRPC	1	Recruiting	NCT03076203

**Table 4:** Selected clinical studies with radium-223 dichloride and combination partners focusing on prostate cancer. Source: https://clinicaltrials.gov/.

Agents	Target	Population	Phase	Status	Identifier
Radium-223 + abiraterone acetate + prednisone	Hydroxyapatite CYP17A	mCRPC	2	Completed, has results	NCT02097303
Radium-223 + enzalutamide	Hydroxyapatite AR	mCRPC	2	Recruiting	NCT02199197
Radium-223 + docetaxel	Hydroxyapatite Tubulin	mCRPC	2	Recruiting	NCT03230734
Radium-223 + olaparib	Hydroxyapatite PARP	mCRPC	1/2	Active, not recruiting	NCT03317392
Radium-223 + niraparib	Hydroxyapatite PARP	mCRPC	1	Active, not recruiting	NCT03076203
Radium-223 + pembrolizumab	Hydroxyapatite PD-1	mCRPC	2	Recruiting	NCT03093428
Radium-223 + atezolizumab	Hydroxyapatite PD-L1	mCRPC	1	Recruiting	NCT02814669
Radium-223 + sipuleucel-T	Hydroxyapatite Immunotherapy	mCRPC	2	Recruiting	NCT02463799
